# Middle Segment-Preserving Pancreatectomy for Recurrent Metastasis of Renal Cell Carcinoma after Pancreatoduodenectomy: A Case Report

**DOI:** 10.1155/2014/648678

**Published:** 2014-06-30

**Authors:** Aiyama Takeshi, Inagaki Mitsuhiro, Akabane Hiromitsu, Yanagida Naoyuki, Shibaki Taiichiro, Shomura Hiroki, Kudo Takeaki, Shonaka Tatsuya, Oikawa Futoshi, Sakurai Hiroharu, Nakano Shiro

**Affiliations:** ^1^Department of Gastroenterological Surgery I, Hokkaido University Graduate School of Medicine, North 15, West 7, Kita-ku, Sapporo 060-8638, Japan; ^2^Department of Surgery, Asahikawa-Kosei General Hospital, 1 Jo 24 Chome 111, Asahikawa 078-8211, Japan; ^3^Department of Pathology, Asahikawa-Kosei General Hospital, 1 Jo 24 Chome 111, Asahikawa 078-8211, Japan

## Abstract

Many cases of surgical resection of metastatic pancreatic tumors originating from renal cell carcinoma have been reported; however, cases of reresection of recurrent pancreatic metastasis of renal cell carcinoma in the remnant pancreas are rare. We performed a second resection for recurrent pancreatic metastasis of renal cell carcinoma six years after pancreatoduodenectomy with pancreaticogastrostomy reconstruction. By performing middle segment-preserving pancreatectomy, we were able to successfully spare the exocrine and endocrine pancreatic function compared to that observed after total pancreatectomy, with no signs of recurrence for two years after the surgery.

## 1. Introduction

Many cases of surgical resection for metastatic pancreatic tumors of renal cell carcinoma have been reported; however, cases of reresection of recurrent pancreatic metastasis of renal cell carcinoma in the remnant pancreas are rare [1, 2]. Recently, a new surgical procedure called “middle segment-preserving pancreatectomy,” which spares the middle portion of the pancreas in order to preserve the exocrine and endocrine pancreatic function, has been reported by Miura et al. [3]. We herein report a case of reresection of recurrent pancreatic metastasis of renal cell carcinoma performed six years after pancreatoduodenectomy with pancreaticogastrostomy reconstruction that successfully preserved the middle portion of the pancreas using distal pancreatectomy.

## 2. Case Presentation

**Figure 1:**
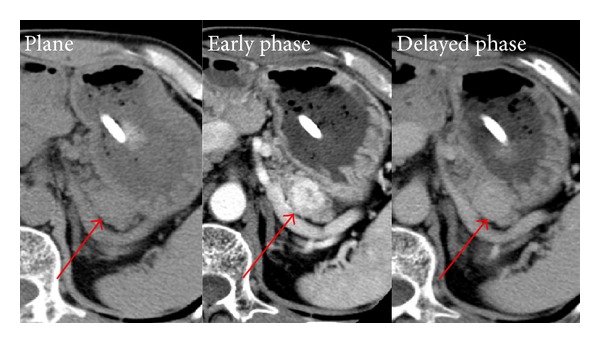
Abdominal computed tomography revealed a hypervascular tumor (arrow) in the middle portion of the pancreas in April 2012.

A 61-year-old male was diagnosed with renal cell carcinoma of the right kidney, which was resected in 1994. In 2005, a single 2 cm hypervascular tumor was detected in the head of the pancreas on computed tomography (CT). We suspected that the lesion was the result of pancreatic metastasis of the renal cell carcinoma and performed pancreatoduodenectomy with pancreaticogastrostomy reconstruction. Informed consent was obtained prior to operation. A pathological examination showed that the resected tumor was a metastatic lesion of renal cell carcinoma (data not shown). In April 2011, another single 2 cm hypervascular tumor was detected in the tail of the pancreas on follow-up CT (Figure 1). Fluorodeoxyglucose-positron emission tomography (FDG-PET) showed no significant FDG accumulation in the tumor of the pancreas compared to the normal pancreatic tissue and no accumulation was detected in other organs (data not shown). The patient had no past history other than that described above and no abnormalities were detected on regular preoperative examinations, including blood tests of the fasting blood sugar and hemoglobin A1c (HbA1c 5.7%) levels. The patient was diagnosed with recurrent pancreatic metastasis of renal cell carcinoma based mostly on his clinical course and preoperative images.

**Figure 2:**
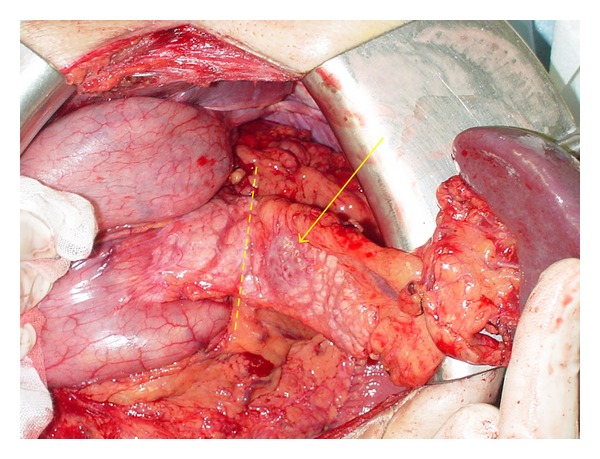
The pancreas was resected approximately 2 cm distal to the tumor (the arrow indicates the tumor and the dashed line indicates the resection line of the pancreas).

The tumor was located 5 cm away from the site of anastomosis of the previous pancreaticogastrostomy. Therefore, we planned to perform distal pancreatectomy in order to preserve the middle portion of the remnant pancreas. The preservation of the spleen was also considered. However the splenic vein was close to the tumor as shown in the CT (Figure 1); we decided to remove the spleen together. In June 2011, we performed laparotomy with an upper median incision and the adhesion was first dissected. The tumor was located 5 cm from the site of anastomosis in the pancreas and stomach; therefore, we decided to preserve the body of the remnant pancreas as planned (Figure 2). After the spleen and tail of the pancreas were mobilized from the retroperitoneum, the splenic artery and vein were ligated and divided at the same level at which the pancreas was transected. The dorsal pancreatic artery was preserved. The remnant pancreas was dissected approximately 2 cm distal to the tumor and the tumor on the tail of the pancreas was resected. The pancreatic resection margin was histologically negative. As a result, approximately 3 cm of the middle portion of the pancreas measured from the site of anastomosis in the pancreas and stomach was preserved. The main pancreatic duct was ligated and the stump of the remnant pancreas was closed, resembling a fish's mouth. The operative time was 145 minutes and the amount of intraoperative blood loss was 107 mL.

**Figure 3:**
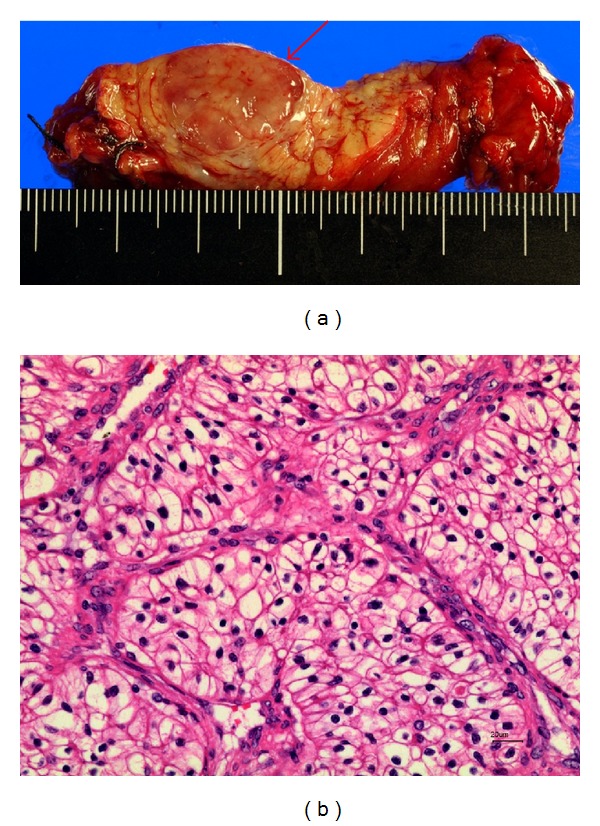
(a) The macroscopic findings showed a single well-circumscribed tumor in the resected specimen. (b) A histological examination revealed metastasis of renal cell carcinoma with the same features as the previously resected specimen (hematoxylin-eosin stain).

The tumor was diagnosed pathologically as reflecting pancreatic metastasis of renal cell carcinoma (Figures 3(a) and 3(b)). The patient's postoperative blood glucose level was well controlled only with oral medicine (the HbA1c level three months after the operation was 6.0% without the use of insulin) and he had no other postoperative complications, such as malabsorption and diarrhea caused by the decrease of exocrine pancreatic function. He was discharged from the hospital on postoperative day 22. Fortunately, after two years of follow-up after surgery, the patient was found to be doing well and had no tumor recurrence.

## 3. Discussion

Resection of metastatic pancreatic tumors accounts for 1-2% of all resections of pancreatic tumors [4, 5]. In addition, 61.7% of metastatic pancreatic tumors are derived from renal cell carcinoma [6]. Saitoh [7] reported that single pancreatic metastases of renal cell carcinoma account for 1% of all metastases of renal cell carcinoma and the number of reports of resection of pancreatic metastasis of renal cell carcinoma is increasing [1]. Evidence-based clinical practice guidelines for treating renal cell carcinoma [8] recommend resection of pancreatic metastasis of renal cell carcinoma, if the metastatic site is resectable and the patient has a good performance status. Indeed, Tanis et al. reported that the 5-year overall survival rate of patients treated with resection who have no extrapancreatic metastasis of renal cell carcinoma is 76%, with a 5-year disease-free survival rate of 60% [1]. There are no randomized controlled trials concerning this issue; however, it is likely that performing resection of pancreatic metastasis of renal cell carcinoma in selected patients contributes to a good prognosis.

These facts suggest that performing reresection of pancreatic recurrence of renal cell carcinoma in well-selected patients may also contribute to improving the prognosis. However, there are few case reports of reresection of the pancreas in patients with pancreatic recurrence of renal cell carcinoma [1, 2]. Tanis et al. [1] reported that, in their study, the recurrence rate in the remnant pancreas in patients with renal cell carcinoma after treatment with pancreatectomy was 4% (12/298) and the median time of recurrence was 42 months and seven of the 12 patients underwent reresection of the remnant pancreas. However, the prognoses of the reresected patients are not available; therefore, the effects of reresection of pancreatic recurrence of renal cell carcinoma are unclear. Our patient has exhibited no recurrence for approximately two years after reresection of pancreatic recurrence of renal cell carcinoma, suggesting that reresection of pancreatic metastasis of renal cell carcinoma can be considered in well-selected patients, for example, those with no other metastases.

In recent years, the number of cases of pancreatic resection of low-grade malignant tumors, such as intraductal papillary mucinous neoplasms and pancreatic endocrine tumors, has been increasing. For low-grade malignant tumors, a new surgical procedure called “middle segment-preserving pancreatectomy,” which preserves the middle portion of the pancreas in order to protect the exocrine and endocrine pancreatic functions, has been reported by Miura et al. [3]. This procedure is associated with several problems, such as a slightly higher rate of postoperative complications than distal pancreatectomy or pancreatoduodenectomy and the need to manage the feeding artery of the middle portion of the pancreas, primarily the dorsal pancreatic artery. However, Cheng et al. [9] reviewed 22 patients who had undergone this procedure and reported that the procedure could serve as a rational choice in well-selected patients to spare the exocrine and endocrine pancreatic functions. Furthermore, preserving the exocrine and endocrine pancreatic functions improves the quality of life compared to that observed after total pancreatectomy. Our case report demonstrates that “middle segment-preserving pancreatectomy” would be a useful surgical procedure for maintaining the quality of life of the patient.
